# Characteristics and outcome of infants with candiduria in neonatal intensive care - a Paediatric Investigators Collaborative Network on Infections in Canada (PICNIC) study

**DOI:** 10.1186/1471-2334-9-183

**Published:** 2009-11-23

**Authors:** Joan L Robinson, H Dele Davies, Michelle Barton, Karel O'Brien, Kim Simpson, Elizabeth Asztalos, Anne Synnes, Earl Rubin, Nicole Le Saux, Charles Hui, Joanne M Langley, Reg Sauve, Louis de Repentigny, Lajos Kovacs, Ben Tan, Susan E Richardson

**Affiliations:** 1 Department of Pediatrics, Stollery Children's Hospital, Edmonton, Alberta, Canada; 2 Department of Pediatrics, Alberta Children's Hospital Calgary, Alberta, Canada; 3 Department of Pediatrics, University of Toronto, Toronto, Ontario, Canada; 4 Department of Pediatrics, Children's & Women's Health Centre of BC, Vancouver, British Columbia, Canada; 5 Department of Pediatrics, Montreal Children's Hospital, Montreal, Quebec, Canada; 6 Department of Pediatrics, Children's Hospital of Eastern Ontario, Ottawa, Ontario, Canada; 7 Department of Pediatrics, IWK Health Centre and Dalhousie University, Halifax, Nova Scotia, Canada; 8 Department of Microbiology and Immunology, Hôpital Sainte-Justine, Montreal, Quebec, Canada; 9 Department of Neonatology, SMBD Jewish General Hospital, McGill University, Montreal, Quebec, Canada; 10 Department of Pediatrics, Royal University Hospital, Saskatoon, Saskatchewan, Canada; 11 Current address: Division of Infectious Diseases, Department of Pediatrics and Human Development, Michigan State University, East Lansing, MI, 48824, USA

## Abstract

**Background:**

There is limited information in the literature on the presentation and prognosis of candidal urinary tract infection (UTI) in infants in the neonatal intensive care unit (NICU).

**Methods:**

This was a prospective cohort study performed in 13 Canadian NICUs. Infants with candidal UTI without extra-renal candidal infection at presentation were enrolled.

**Results:**

Thirty infants fit the study criteria. Median birth weight and gestational age were 2595 grams (range 575-4255) and 35 weeks (range 24-41) with 10 infants being < 30 weeks gestation. The most common primary underlying diagnosis was congenital heart disease (n = 10). The median age at initial diagnosis was 16 days (range 6-84 days). Renal ultrasonography findings were compatible with possible fungal disease in 15 of the 26 infants (58%) in whom it was performed. Treatment was variable, but fluconazole and either amphotericin B deoxycholate or lipid-based amphotericin B in combination or sequentially were used most frequently. Extra-renal candidiasis subsequently developed in 4 infants. In 2 of these 4 infants, dissemination happened during prolonged courses of anti-fungal therapy. Three of 9 deaths were considered to be related to candidal infection. No recurrences of candiduria or episodes of invasive candidiasis following treatment were documented.

**Conclusion:**

Candidal UTI in the NICU population occurs both in term infants with congenital abnormalities and in preterm infants, and is associated with renal parenchymal disease and extra-renal dissemination. A wide variation in clinical approach was documented in this multicenter study. The overall mortality rate in these infants was significant (30%). In one third of the deaths, *Candida *infection was deemed to be a contributing factor, suggesting the need for antifungal therapy with repeat evaluation for dissemination in infants who are slow to respond to therapy.

## Background

Isolation of *Candida *from the urine of newborns can be indicative of contamination or of urinary tract infection. Although bacteremia is a complication of less than 3% of pediatric nosocomial bacterial urinary tract infections (UTIs) [1], it is not clear how often candidal UTI is a precursor to candidemia or to candidal infection at other sites. The primary purpose of this multi-center study was to describe the presentation, therapy, and prognosis of candidal UTIs in infants in the neonatal intensive care unit (NICU) in the absence of documented extra-renal infection at presentation.

## Methods

This prospective study was performed in 13 tertiary level NICUs in 9 cities in Canada by members of the Paediatric Investigators Collaborative Network on Infections in Canada (PICNIC). The study protocol was approved by the institutional ethics review board at each center. As part of a larger study [2], infants ≤ 90 days of age were prospectively enrolled between February 1, 2001 and July 31, 2003 if they met one of three criteria: 1) *Candida *was isolated from any sterile body site, 2) there was histologic or ophthalmologic evidence of *Candida *infection, or 3) they had candidal UTI, defined as growth of *Candida *from urine at > 10^6 ^CFU/L from a suprapubic aspirate or > 10^7^CFU/L from a bladder catheter specimen (2). In this report we describe those infants who fulfilled the third criterion without documented evidence of extra-renal infection at the time of enrolment. Infants with *Candida *isolated only from a bag urine were excluded. Institutions were instructed to follow their usual protocols for the diagnosis and treatment of candidal infection during the study.

Demographic and clinical data were collected on a standard case report form and entered into an Access (Microsoft Access 2002) database. Data were analyzed using SAS (version 9.1, SAS Institute Inc, Cary, NC, USA.). The day the first positive urine culture with significant growth of *Candida *was submitted was considered to be the day of identification of candiduria, recognizing that the onset of candiduria may have been earlier in some cases. The local investigator was asked to determine if *Candida *infection contributed to death.

## Results

### Demographics and prior use of antifungal therapy

Thirty infants (16 males, 14 females, 23 singletons and 7 twins) met the study inclusion criteria (Table 1). The median birth weight was 2595 grams (range 575 - 4255 grams) and the median gestational age (GA) was 35 weeks (range 24 - 41 weeks). Ten infants (33%) were born prior to 30 weeks. Delivery was vaginal (n = 13), via elective cesarean section (n = 6) or via emergency cesarean section (n = 11). The primary reason for NICU admission was congenital heart disease (n = 10, of which 7 had a GA = 37 weeks), respiratory distress (n = 8), renal disease (n = 5), sepsis (n = 3), gastrointestinal disease (n = 2), and trisomy 21 (n = 2). None of the infants received prophylactic topical or systemic antifungal therapy. Thirteen infants (43%) received systemic corticosteroids for 1 to 31 days (median 3 days) prior to the candidal UTI. These consisted of a wide range of doses of intravenous methylprednisolone, hydrocortisone, or dexamethasone.

**Table 1:** Characteristics and outcome of 30 infants with candiduria from 13 Canadian neonatal intensive care units

Case	GA	BW	Primary Diagnoses	Corticosteroids prior to candiduria	Day of life candiduria detected (Source)	Species	Sites of extra-renal dissemination	Outcome
1	24	780	Trisomy 21, Prematurity	yes	19 (catheter)	*C. albicans*	None	deceased

2	24	752	RDS, Prematurity	yes	16 (catheter)	*C. glabrata*	None	

3	33	2435	Renal Disease	no	13 (catheter)	*C. albicans*	None	

4	35	3320	Sepsis	no	17 (catheter)	*C. parapsilosis*	None	

5	41	3385	RDS, Renal Disease	yes	6 (catheter)	*C. albicans*	None	

6	35	2570	Renal Disease	no	15 (SPA)	*C. albicans*	None	

7	38	3000	Omphalocele	yes	11 (catheter)	*C. albicans*	None	

8	26	635	CHD, Prematurity	no	29 (catheter)	*C. parapsilosis*	None	deceased

9	34	1875	Sepsis	no	15 (SPA)	*C. parapsilosis*	None	

10	40	2800	CHD	no	15 (catheter)	*C. lusitanae*	None	

11	27	1025	RDS, Prematurity	yes	22 (SPA)	*C. albicans*	None	

12	40	3024	CHD	yes	39 (catheter)	*C. parapsilosis*	None	

13	37	3090	Renal Disease	no	15 (catheter)	*C. albicans*	None	

14	39	3400	CHD	no	16 (catheter)	*C. tropicalis*	None	deceased

15	38	4255	GI Disease	no	14 (catheter)	*C. albicans*	None	

16	35	2620	Renal Disease	yes	21 (SPA)	*C. albicans*	None	

17	40	3630	CHD	yes	11 (catheter)	*C. albicans*	None	

18	38	2750	Trisomy 21	yes	35 (catheter)	*C. albicans*	None	deceased

19	40	3500	CHD	yes	15 (catheter)	*C. tropicalis*	None	deceased*

20	39	3930	CHD	no	84 (catheter)	*C. albicans*	None	deceased

21	40	3030	Renal Disease	no	11 (SPA)	*C. albicans*	None	

22	30	1445	Sepsis	no	7 (catheter)	*C. albicans*	None	

23	25	815	RDS, Prematurity	yes	48 (catheter)	*C. lusitanae*	None	

24	24	650	RDS, Prematurity	no	9 (SPA)	*C. albicans*	None	

25	25	725	RDS, Prematurity	no	35 (catheter)	*C. albicans*	None	

26	24	575	RDS, Prematurity	yes	56 (catheter)	*C. albicans*	None	

27	26	830	Prematurity, NEC	no	6 (SPA)	*C. albicans*	Blood	

28	28	1170	CHD	no	58 (catheter)	*C. albicans*	Brain	deceased*

29	33	2200	CHD	no	77 (SPA)	*C. parapsilosis*	Blood	deceased

30	37	3225	CHD	yes	50 (catheter)	*C. albicans*	Blood	deceased*

BW - birthweight; CHD - congenital heart disease; GA - gestational age; NEC - necrotizing enterocolitis; RDS - respiratory distress syndrome; SPA - suprapubic aspirate

* Death was thought to be related to candidal infection

### Extra-renal dissemination of candidal infection

Three of the 30 infants developed candidemia 2 to 41 days following the candidal UTI and a fourth infant had evidence of central nervous system candidal infection first detected at autopsy (Table 2).

**Table 2:** Infants who developed extra-renal dissemination of candidal infection from a cohort of 30 infants diagnosed with candiduria

Patient number	GA	Primary diagnosis	Extra-renal site	Species	Days between positive urine culture and positive culture at extra-renal site	**Cultures performed between date of positive urine and date of positive culture at extra-renal site**^**1**^	Therapy between candiduria and positive culture at extra-renal site	Outcome of candidal infection	Treatment after diagnosis of extra-renal candidal infection
1	26 weeks	Prematurity	Blood	*C. albicans*	2	1 urine culture positive for *C. albicans*	None	Survived	32 days AMP, FCZ and 5FC in various combinations

2	28 weeks	Congenital heart disease	CNS (at autopsy)^2^	*C. albicans*	11	4 negative blood cultures	7 days AMP	Died	None

3	33 weeks	Congenital heart disease	Blood	*C. parapsilosis*	41	5 negative urine cultures^3^	13 days AMP	Survived^4^	28 days AMP and L-AMP

4	37 weeks	Congenital heart disease	Blood	*C. albicans*	32	5 urine cultures positive for *C. albicans *and 4 negative blood cultures	26 days AMP, FCZ or both^5^	Died	4 days L-AMP and FCZ

AMP - amphotericin B deoxycholate; CNS - central nervous system; FCZ - fluconazole; GA - gestational age; L-AMP- lipid-based amphotericin B; 5FC - 5 flucytosine

^1^All had a negative blood culture on the day of diagnosis of candiduria

^2^Prior to the positive urine culture, had 7 negative blood cultures and 2 negative CSF cultures. Between the onset of candiduria and death had 4 negative blood cultures but cerebrospinal fluid not obtained

^3 ^Blood culture was not performed initially or until 41 days after the date of the positive urine culture

^4^Ultimately died of congenital heart disease

^5^There was a 5-day gap after the first 5 days of therapy.

### Clinical and laboratory features on the day of diagnosis of candidal UTI

Candidal UTI was diagnosed at a median of 16 days of age (range 6 to 84 days). Three infants of GA 26, 30, and 41 weeks had possible congenital candidal infection with diagnosis on days 6, 7, and 6 of life, respectively. The placenta was examined for these 3 infants. The one from the infant born at 41 weeks GA was normal while the 2 preterm infants showed chorioamnionitis and funisitis.

Clinical findings on the day of diagnosis included fever of 38.0°Celsius or higher (n = 7, 23%), feeding intolerance (n = 2, 7%), and respiratory deterioration (n = 5, 17%). Rash occurred on the day of diagnosis in all 3 infants with possible congenital candidiasis (diaper dermatitis in the preterm infants and a generalized excoriating rash in the term infant). Three other infants born at 25, 27, and 35 weeks GA had diaper dermatitis when they presented on days 48, 22, and 21 of life

Positive urine cultures were obtained via suprapubic aspirate (n = 8) or bladder catheter (n = 22). Seventy percent were *C. albicans *(Table 3). Urinalysis was obtained within 24 hours of diagnosis of candidal UTI for only 10 infants. Two infants had no pyuria, 2 had an occasional WBC/HPF, 2 had 1-10 WBCs/HPF, and 4 had >10 WBCs/HPF. Hematologic abnormalities included a peripheral leukocyte count of <5 × 10^9^/L in 1 infant and >25 × 10^9^/L in 2 infants, an absolute granulocyte count of >10 × 10^9^/L in 10 infants, and platelets <100 × 10^9^/L in 6 infants. Serum creatinine at diagnosis was <60 μmol/L in 16 infants, 61-100 μmol/L in 5 infants, 101-150 μmol/L in 3 infants, >150 μmol/L in 4 infants and not recorded in 2 infants.

**Table 3:** Species of *Candida *in 30 infants in neonatal intensive care units with candiduria

Species	**Number of cases of candiduria (%) - current study; ** **n = 30**^**1**^	Number of cases of candiduria (%) (3) n = 25	Number of cases of candiduria (%) (4) n = 36^2^
Years of study	2001-2003	1989-1995	1982-1993

*Candida albicans*	21 (70%)	15 (60%)	26 (72%)

*Candida parapsilosis*	4 (13%)	8 (32%)	7 (19%)

*Candida tropicalis*	2 (7%)	2 (8%)	1 (3%)

*Candida lusitaniae*	2 (7%)	0	0

*Candida glabrata*	1 (3%)	0	2 (6%)

^1^Two of the infants with *C. albicans *and one with *C. parapsilosis *were fungemic.

^2 ^Study reports 41 infants but microbiologic data only provided for 36 infants

Blood culture was performed and was negative for fungi in 24 of the 30 infants on the day of diagnosis of candidal UTI and in another 2 infants prior to initiation of antifungals. Of the four infants who did not have blood cultures prior to antifungals, three subsequently had no clinical or laboratory evidence of invasive candidal infection but one had a positive blood culture 41 days later (Table 1). Lumbar puncture was performed in only 5 infants within 2 days of the diagnosis of candidal UTI. A cell count was available in 3 of these infants, of whom 2 had pleocytosis (WBC 44 × 10^6^/L (100% monocytes) with RBC 62 × 10^6^/L, and WBC 315 × 10^6^/L (45% polymorphonuclear cells) with RBC 12,720 × 10^6^/L) and all 5 CSF cultures were sterile. Four other infants had CSF obtained more than 2 days after the diagnosis of UTI but prior to laboratory documentation of clearance of candiduria. All CSF cultures were sterile, and there was no pleocytosis in the 2 cases where a cell count was available.

### Radiographic findings

Renal ultrasonography revealed abnormalities compatible with fungal disease in 15 of the 26 infants (58%) in whom it was performed. Twenty-four of the 26 scans were performed within 4 days of the onset of candiduria. These abnormalities included diffuse parenchymal echogenicity (n = 5), focal parenchymal echogenicity (n = 1), unspecified echogenicity (n = 1), dilatation of the collecting ducts (n = 3) and both parenchymal echogenicity and dilatation of the collecting ducts (n = 5). Almost all abnormalities were bilateral. The radiologic appearance was consistent with "fungal balls" in 3 of these 15 infants - a term infant with diffuse parenchymal disease and 2 infants born at 25 and 35 weeks GA with dilated collecting ducts, none of whom developed candidemia. Follow-up imaging was not performed in these 3 infants.

Ultrasonography of the liver and spleen was abnormal in 6 of the 16 infants (37.5%) in whom it was performed, revealing hepatomegaly (n = 2), a solitary hepatic hemangioma, a calcified hepatic thrombosis, a splenic hematoma, and diffusely abnormal splenic echogenicity of uncertain significance. None of the findings were considered to be suggestive of hepatosplenic candidiasis.

### Ophthalmologic findings

None of the 12 infants who had fundoscopic examinations had evidence of fungal retinitis.

### Therapy

**Figure 1:**
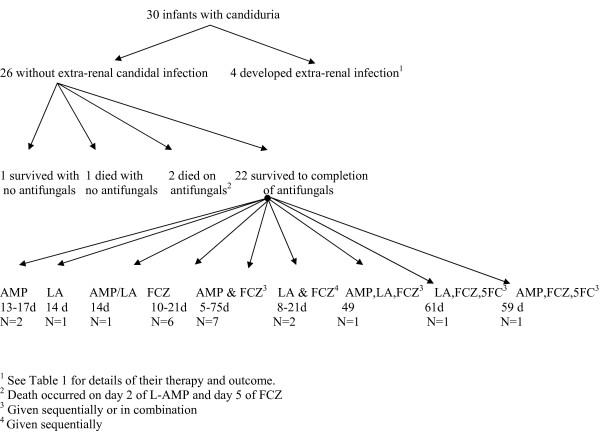
**Antifungal therapy offered to 30 infants with candiduria in neonatal intensive care units**. AMP - amphotericin B deoxycholate; d-days; FCZ - fluconazole; L-AMP- lipid-based amphotericin B; 5FC - 5 flucytosine.

Two infants did not receive antifungal therapy - a term infant with renal failure who was treated for a *Pseudomonas *UTI and had no follow-up urine cultures prior to discharge (patient 13 in Table 1) and an infant with trisomy 21 who had candidal UTI 3 days prior to death with no evidence of *Candida *infection at autopsy (patient 18 in Table 1). Therapy for the remaining infants consisted of fluconazole (FCZ), amphotericin B deoxycholate (AMP), lipid-based amphotericin B (L-AMP) and 5-fluctyosine (5-FC) in various combinations (Figure 1). For the 22 infants who survived to the end of therapy and did not develop extra-renal candidal infection, 4 infants received AMP or L-AMP alone, 6 infants received FCZ alone, 10 infants received a combination of AMP or L-AMP and FCZ and 2 infants received AMP or L-AMP with FCZ and 5-FC. The median total duration of antifungals in these 22 infants was 16.5 days (range 5-75 days). For the 3 infants with suspected renal fungal balls, one of the term infants received just two days of L-AMP prior to death (Table 1) while the 2 survivors were treated for 14 and 75 days with sequential AMP and FCZ.

### Complications of Therapy

A clinically significant rise in creatinine (defined as an increase of ≥ 20% to a level ≥60 μmol/l) was documented in 5 infants while on therapy (a rise from 142 to 178 μmol/L on day 3 of AMP, a rise from 171 to 310 μmol/L on day 9 of L-AMP following a 3 day course of AMP, a rise from 104 to 137 and from 36 to 87 μmol/L on days 2 and 5 of L-AMP respectively and a rise from 32 to 98 μmol/L on day 5 of FCZ). For the 3 infants with suspected fungal balls, the highest serum creatinine levels were 66, 91, and 97 μmol/L respectively. Hypokalemia (defined as serum potassium < 3.3 mEq/L) was documented in 10 infants (33%) of whom 4 were born prior to 30 weeks GA. Attributing hypokalemia to specific antifungals was not possible as 9 of these infants were treated with sequential or combination drugs. New-onset thrombocytopenia (platelets <100 × 10^9^/L) was documented after commencing antifungals in 5 infants (17%). Four infants developed hypotension while on antifungal therapy (two on FCZ and one on each of AMP and L-AMP).

### Outcome

There were 9 deaths among the 30 infants in the study (30%), all in infants with significant underlying conditions (7 with congenital heart disease and 2 with trisomy 21). In three of these deaths, *Candida *infection was thought to be a contributing factor. Two of the deaths were in infants who developed disseminated disease (patients 2 and 4 in Table 1), and one occurred in an infant without evidence of extra-renal candidal infection. The latter infant was born at 40 weeks GA with congenital heart disease and died following 2 days of therapy for candidal UTI, with suspected renal fungal balls on renal ultrasonography. No autopsy was performed. Five of the 6 other infants who died had autopsies, none of which revealed evidence of candidal infection. No recurrences of candiduria or isolation of *Candida *from a sterile site were documented prior to hospital discharge among the 21 survivors, but 5 infants did not have follow-up urine cultures obtained.

## Discussion

Multiple previous studies outline the epidemiology and clinical course of invasive candidiasis in the NICU, but there have been limited studies of candidal UTI in the absence of extra-renal disease as detected by routine investigations. This report describes 30 such cases presenting to 13 Canadian NICUs over a 30-month period, many in term or near-term infants with major congenital abnormalities of the heart or kidneys. In addition our results suggest a lack of distinguishing clinical or laboratory features at diagnosis, a high rate of abnormalities on renal ultrasonography (>50%), and a significant proportion (one third) of the total mortality related to *Candida *infection.

Our finding of candidal UTI in term infants with congenital anomalies differs from two previously published studies of candidal UTIs in NICUs. Philips *et al *used an identical definition for candidal UTI to the current study but only enrolled infants 7 days of age or older (n = 25) [3]. The second study by Bryant *et al *enrolled infants from birth (n = 41) but accepted any growth of *Candida *from a catheterized urine as being indicative of a UTI [4]. These previous studies described candidal UTIs mainly in very low birth weight infants (median gestational age 26 weeks [3] and 27 weeks [4] versus 35 weeks in the current study). This discrepancy is likely due to our exclusion of infants who had candidemia on the day of diagnosis of candidal UTI. In the cohort of 66 infants with systemic candidiasis that were part of our larger study who met the first inclusion criteria but are not included in the current report, 8 infants had both blood and urine cultures positive for *Candida *and their median gestational age was 25.5 weeks (unpublished data), consistent with these previously published studies. Differences in NICU populations between the studies may also be a factor with the current multicenter study including a higher proportion of term infants with congenital heart disease than many studies of single NICUs. Although there are no previous studies looking at candidal UTI in term or near-term infants in NICU, a study that looked at invasive candidiasis in infants with a birth weight over 2500 grams described 13 of 17 infants (76%) with serious congenital anomalies [5]. This pattern fits with the current study where two-thirds of the infants were born after 29 weeks gestation and over half had serious congenital anomalies. In a study from the United Kingdom of infants with fungal infections and a birthweight < 1500 grams, 26 of 94 cases had funguria with 6 having isolated funguria [6]. The species of *Candida *were comparable in all NICU candidal UTI studies to date (Table 2).

On renal ultrasonography, parenchymal changes predominated in the current study, suggesting the possibility of unrecognized hematogenous spread of *Candida *in infants who are suspected to have candidal infection limited to the urinary tract. Ascending infection would be expected to result in isolated pelvicalyceal disease, with only 3 of the 15 abnormal renal ultrasounds fitting this pattern. However, the terminology for renal ultrasonography reporting in neonates is not uniform. Renal fungal balls or abscesses were suspected in 35% [3] and 42% [4] of the 55 infants with renal imaging or autopsy diagnoses in the previous studies. In the current study, only 12% of the 26 infants with renal imaging had a fungal ball mentioned in the report, but many had changes that appeared to be consistent with those reported as fungal balls in a previous study [4]. Renal fungal balls can be confused with fibrin, blood clots, necrotic papillae, nephrocalcinosis, or tumors on renal ultrasonography [7] so that the interpretation may be influenced by the information provided to the radiologist about the possibility of candidal infection. In one study, about half of the suspected fungal balls were apparent only on follow-up ultrasounds [4] which were not routinely performed in the current study.

Although surgical intervention for renal fungal disease in the collecting system has been described in numerous case reports [7], it was not required for infants with fungal balls in our study. For the 66 infants with candidal UTI described in the two previous case series [3,4]22 infants had suspected fungal balls with 2 having partial obstruction but surgery was required for only one infant with a renal abscess [4]. In another recent study of 9 infants with suspected renal fungal balls, surgical management was not required [8]. This suggests that medical management can be anticipated to be successful in the majority of cases, even in the presence of documented fungal balls on imaging, unless there is concomitant total obstruction.

The need for, choice and duration of antifungal treatment for candidal UTI in the absence of extra-renal disease has not been studied and there are no widely-accepted guidelines, explaining the marked variation in therapy in the current study. Two of the 3 deaths that were thought to be related to candidal infection were a result of dissemination of disease, indicating that cases with "apparent isolated candiduria" may later disseminate or may have undetected foci of infection at non-renal distant sites. The significant rate of extra-renal dissemination (13.3%) supports the use of systemic antifungal therapy when candiduria occurs with a significant colony count in the NICU.

Most infants with candidal UTI in previous reports were successfully treated with AMP or FCZ, typically given for a minimum of 7 days after urine cultures became sterile [7]. Much longer courses have often been given if changes are noted on renal ultrasonography, but it appears that there is no need to document resolution of these changes prior to stopping therapy [7] There were only 2 cases of suspected treatment failure in the current study where death was attributable to candidal infection despite 7 or more days of appropriate therapy, both in infants with extra-renal dissemination (Table 1). There were no recurrences of candiduria, suggesting that any of the multiple regimes used by clinicians for candidal UTI are likely to be successful if extra-renal invasive candidal infection has been excluded. Two of the three cases of candidemia occurred after long courses of antifungals, suggesting that extra-renal candidal infection should be sought even in infants on treatment with a slow response to therapy. The role of parenteral prophylactic antifungals for candiduria could not be addressed in the current study as they were not used in any of the NICUs.

The primary limitation in drawing conclusion from this study is that although the patients were enrolled prospectively, investigations for dissemination were at the discretion of the attending physician. This resulted in not all patients being consistently evaluated for meningitis, retinitis, renal parenchymal disease, recurrent candiduria, or even candidemia. However, we recognize that false-negative blood and CSF cultures occur frequently in neonatal candidasis so associated morbidity is not always recognized even when infants are fully evaluated for disseminated disease. Nonetheless, even though it is widely accepted that all infants with candidemia should be investigated for end-organ damage [7], the need for full investigation of infants with candiduria in the absence of candidemia is less clear from the previous literature. The role of fundoscopy in infants with candiduria alone is not clear although a study has shown a higher incidence of candidial retinitis with candidaemia of greater gestational age [9], suggesting that fundoscopy is indicated even in term infants with extra-renal candidiasis.

Further limitations are that it would have been ideal to have all renal ultrasounds interpreted by a single radiologist, and that changes in management of infants will have occurred since this study was performed. For example, fewer infants would be exposed to post-natal corticosteroids and echindocandins are now used as antifungal therapy in some centers.

One of the limitations of all studies to date is that definitions devised for the diagnosis of bacterial UTIs have been extrapolated to fungal UTIs, without validation of these definitions in any age group [1]. There are no standard definitions for colony counts defining UTIs in children with indwelling bladder catheters [1] which would include a small number of infants in the current study. The fact that over half of the renal ultrasounds in our study demonstrated abnormalities consistent with fungal infection of the renal parenchyma or collecting system suggests that the specificity of the definitions used is reasonable. It is however possible that clinically significant fungal UTIs can occur at lower colony counts than those applied in this study, as has been described in infants with suspected fungal balls [10]. In addition, infants with fungal UTI may have been missed if only a bag urine had been submitted or if antifungals had been started prior to obtaining the urine specimen.

## Conclusion

In this study of Canadian neonates, we were able to show that neonates presenting as candidal UTI without dissemination, within the NICU population of low birth weight infants and older gestational age infants with underlying illness, is associated with significant morbidity and mortality. It is difficult to separate out the effect of candiduria on outcome versus the tendency of infants with a poor prognosis to develop candiduria. There is significant variation in the diagnosis and management of candiduria in academic tertiary level NICUs in Canada. The cases of extra-renal invasive candidal disease suggest that investigation for *Candida *in the blood, cerebrospinal fluid, renal parenchyma and retina may be indicated along with systemic antifungal therapy. Evidence of extra-renal spread in the face of appropriate systemic therapy may be a poor prognostic sign, and is not restricted to extremely premature infants. Multi-center randomized trials are needed to determine the optimal therapy and duration of treatment for this relatively rare entity.

## Competing interests

Anne Synnes owns shares in Al-Pharma which manufactures amphotericin B.

## Authors' contributions

SER wrote the initial protocol with revisions then provided by HDD and JLR. All authors with the exception of MB contributed cases to the study. MB performed the data analysis. JLR wrote the manuscript with revisions provided by all authors. All authors read the final manuscript.

## Pre-publication history

The pre-publication history for this paper can be accessed here:

http://www.biomedcentral.com/1471-2334/9/183/prepub
